# Supplementary material for: Radiolabeling, Quality Control and In Vivo Imaging of Multimodal Targeted Nanomedicines
Source: Pharmaceutics. 2022 Dec 1;14(12):2679. doi: 10.3390/pharmaceutics14122679 (PMC9784797; doi:10.3390/pharmaceutics14122679)
Supplement: Supplementary file 1 [file pharmaceutics-14-02679-s001.zip › Supplementary Materials.pdf]

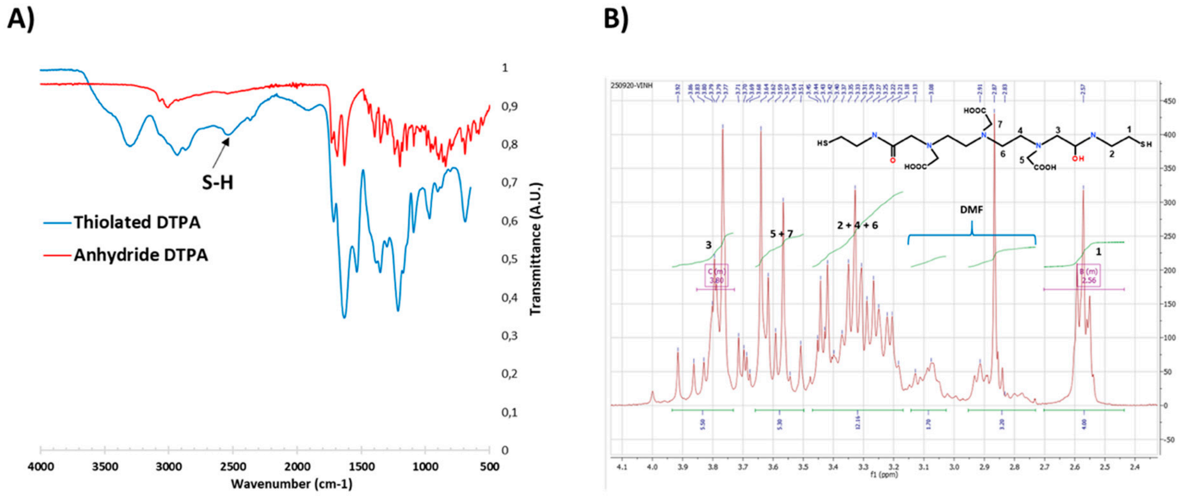

Fig.S1. FT-IR and ^{1}H-NMR spectra of thiolated DTPA. A) FT-IR spectrum of DTPA-SH vs bi-anhydride DTPA; B) ^{1}H-NMR in D_2O spectrum of DTPA-SH

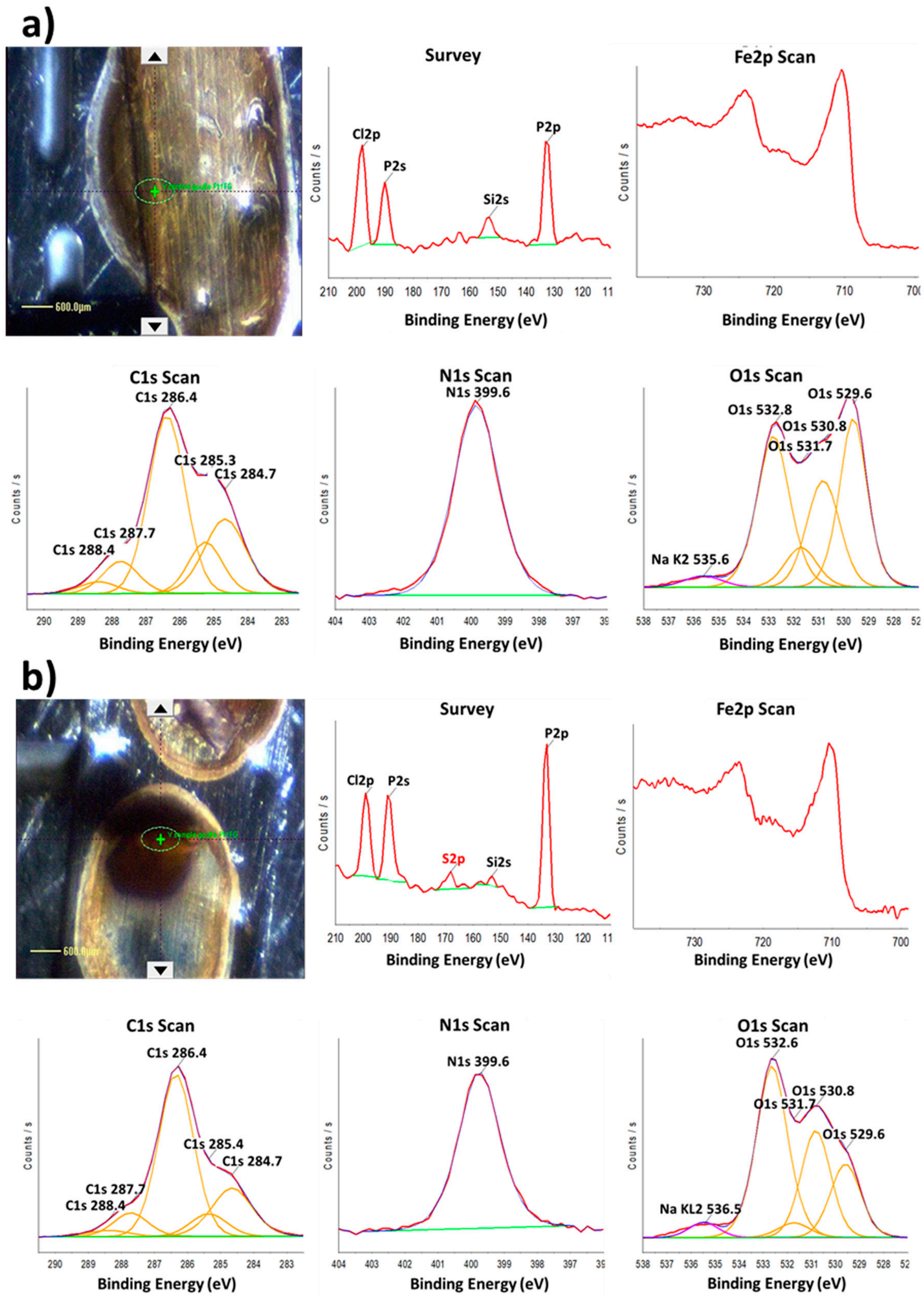

Fig.S2. Optical images of the analyzed area by XPS, part of survey focusing on S2p peak, Fe2p spectrum, and fitted C1s, O1s, N1s XPS spectra on NV-scFv (a) and NV-scFv-DTPA$_{0.6}$ (b) sample drops.

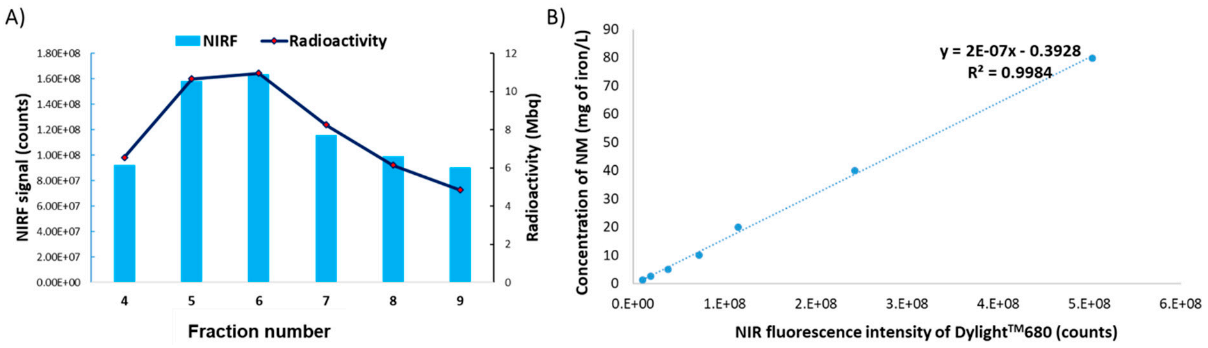

Fig.S3. A) Elution profiles of six fractions the most concentrated of NM-scFv-DTPA-^{111}In after PD-10 column purification according to their NIRF intensity (histogram) and their radioactivity (curve); B) Calibration range of NM-scFv-DTPA (mg of iron/L) according to the NIRF intensity of Dylight^{TM}680 used for the calculation of injected formulation (Dilution factor: 15 µL of NM-scFv-DTPA diluted in 35 µL of PBS 1X).
