# Supplementary figures and images for: Radiolabeling, Quality Control and In Vivo Imaging of Multimodal Targeted Nanomedicines
Source: Pharmaceutics. 2022 Dec 1;14(12):2679. doi: 10.3390/pharmaceutics14122679 (PMC9784797; doi:10.3390/pharmaceutics14122679)

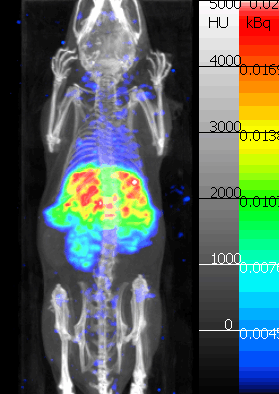

Supplement: Supplementary file 1 [file pharmaceutics-14-02679-s001.zip › Video S1.A1.gif]

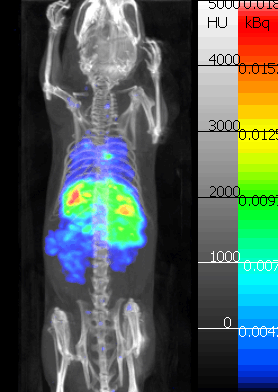

Supplement: Supplementary file 1 [file pharmaceutics-14-02679-s001.zip › Video S1.A2.gif]

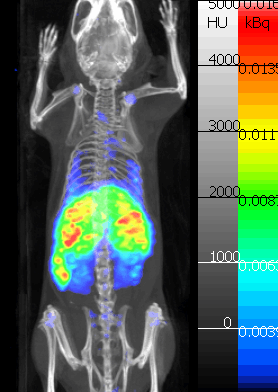

Supplement: Supplementary file 1 [file pharmaceutics-14-02679-s001.zip › Video S1.A3.gif]

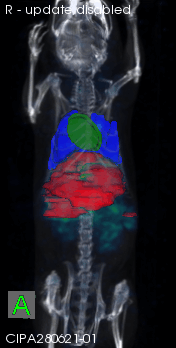

Supplement: Supplementary file 1 [file pharmaceutics-14-02679-s001.zip › Video S1.B.gif]
